# Improving Health Equity by Screening for Poverty: A Survey of Family Physician Screening Behaviors and Perceptions in Toronto, Canada

**DOI:** 10.1089/heq.2021.0074

**Published:** 2022-02-14

**Authors:** Curtis Chin, Michelle Amri, Michelle Greiver, Kimberly Wintemute

**Affiliations:** ^1^ Department of Emergency Medicine, Cape Breton Regional Hospital, Sydney, Canada.; ^2^ Dalla Lana School of Public Health, University of Toronto, Toronto, Canada.; ^3^ Takemi Program in International Health, Department of Global Health and Population, Harvard T.H. Chan School of Public Health, Harvard University, Boston, USA.; ^4^ Department of Family and Community Medicine, North York General Hospital, North York, Canada.; ^5^ Department of Family and Community Medicine, Temerty Faculty of Medicine, Toronto, Canada.

**Keywords:** health equity, social determinants of health, family physicians, primary care, poverty screening, family health team

## Abstract

**Purpose:** Given the importance of socioeconomic status in both directly and indirectly influencing one's health, “poverty screening” by family physicians (FPs) may be one viable option to improve patient health. However, rates of screening for poverty are low, and reported barriers to screening are numerous. This study sought to collate and investigate reasons for refraining from screening among FPs, many of whom had opted into a Targeted Poverty Screening (TPS) Program, to be able to enhance uptake of the intervention. The TPS Program is a “targeted screening and referral process,” whereby medical charts of adult patients residing in “deprived neighborhoods,” as determined by postal code, were flagged for screening for FPs who elected to partake in the program.

**Methods:** A survey containing 15 questions was developed through an iterative process with pilot-testing by faculty physicians. The survey was administered to FPs registered in the North York Family Health Team (NYFHT) using Qualtrics© research software.

**Results:** Half of the respondents (*n*=19/38; 50%) indicated that they enrolled in the TPS program. Irrespective of enrollment in the TPS Program, the majority of respondents (*n*=31/38; 81.6%) stated that they elect to screen their patients for poverty using the evidence-based question of “do you have difficulty making ends meet at the end of the month?.” Among those not enrolled in the program, 84.2% (*n*=16/19) of respondents indicated that they screened their patients for poverty and 15.8% (*n*=3/19) indicated they did not. Among respondents who said they did not screen (*n*=7/38; 18.4%), the reasons for not screening patients were as follows: forgot (*n*=2; 28.6%); time constraints/feel uncomfortable asking (*n*=1; 14.3%); and “feel I know patients well” (*n*=1; 14.3%). For the remaining respondents, a nurse or locum did the screening as part of a periodic health review (i.e., patient was screened, but not by the FP completing the survey (*n*=3).

**Conclusion:** This study yielded numerous insights, such as barriers faced by FPs in undertaking poverty screening that differs from the literature. The findings suggest that (1) barriers faced by FPs in poverty screening can be mitigated, (2) there is a need to integrate screening into routines and normalize the activity, and (3) there is a need for enhanced training to support patients of lower socioeconomic status.

## Introduction

*“My wish for the future of health care in Canada? I naïvely hope to never have to write ‘poverty’ as a contributing cause of death, ever.”^1^ – Dr. Buchman, Past President, Canadian Medical Association*

Socioeconomic status is a social determinant of health (SDH): the “broad range of personal, social, economic, and environmental factors that determine individual and population health”.^2^ Poverty and other SDH are complexly related to one another.^3^ As poverty is a cause of ill health, “poverty screening” may be a viable approach to address aspects of poor health that are associated with one's socioeconomic status. This can be compared to tobacco, where documenting patient risk is a crucial first step to be able to integrate public health measures to clinical practice.^4^

This information around a patient's socioeconomic status can then be used to develop novel interventions, identify and address individual gaps in care (e.g., because those with lower incomes have less access to specialists, the family physician (FP) can focus attention on ensuring that this is not the case for their patients),^5^ refer patients to a case worker whose job is to optimize income for the patients referred (e.g., by applying for income supplementation programs and linking patients to community resources), among others.

The ultimate goal of screening is to identify patients who may benefit from an intervention before the “disease” is clinically evident. Wilson's original criteria provided a good framework for screening programs.^6^ As per the updated 2008 criteria from the World Health Organization, some (but not all) of the criteria are the following: “The screening program should respond to a recognized need; There should be a defined target population; There should be scientific evidence of screening program effectiveness; The overall benefits of screening should outweigh the harm”.^7^ This guides some of the long-term goals of this project, that is, identifying a target population (e.g., certain postal codes); responding to a need (e.g., low-income); and having overall benefits outweigh the harm (e.g., embarrassment).^7^

With 10.1% of Canada's population living below the poverty line (determined based on a market basket measure), this is a widespread issue that needs consideration when providing health care.^8^ An example of a validated poverty screen is the question: “Do you have difficulty making ends meet at the end of the month?” which has been identified as a good predictor of poverty through assessment of 156 questionnaires completed by primary care patients and correlated with demographic data, whereby poverty was calculated; along with two additional questions around food and housing security (explored in the discussion).^9^

In addition to other professions, FPs are well-positioned to (1) address public health issues; (2) take a holistic view of a patient (including social factors and socioeconomic status); (3) have less loss to follow-up and stronger rapport with patients; and (4) preemptively address issues (i.e., activate preventive healthcare measures).^1^ One such opportunity to improve health may be in the form of screening for poverty, aimed at addressing health conditions which arise from lower socioeconomic status.

Despite the convenient position of FPs—and despite interventions designed to address the issues that come with poverty—rates of screening are low.^10–13^ Screening for poverty may increase targeted interventions, which may in turn begin to address associated issues on a patient-by-patient basis. Identifying barriers to screening (so as to address them) is a crucial step. As such, further research around primary health care teams' screening and intervention practices with regard to poverty is sorely needed.^1^

In North York, one of the six administrative districts of Toronto, Canada, a clinician-led program was developed to provide training on screening and managing poverty to FPs in the North York Family Health Team (NYFHT), which included 86 FPs at the time of the study. This Targeted Poverty Screening (TPS) Program is a “targeted screening and referral process,” whereby medical charts of adult patients residing in “deprived neighborhoods,” as determined by postal code, were flagged for screening for FPs who elected to partake in the program.^13^

Despite this pilot study being “opt-in” (all participants chose to participate), data^13^ suggested that only 25% of patients who were flagged as potentially low-income by postal code mapping were screened at an appointment over a six-month period. The present study was designed to explore FPs' barriers to screening for poverty.

## Methods

### Survey design

A survey was developed through an iterative process. Drafts of the survey were reviewed by two faculty physicians for clarity, brevity, and relevance. The survey was designed to gather demographic data and review FPs' perceptions about screening for poverty and contained both open- and closed-ended questions (Appendix A1). The research protocol was approved by the Toronto Academic Health Sciences Network Research Ethics Board.

### Survey dissemination and data collection

The survey was designed using Qualtrics© research software and distributed by email to all FPs currently practicing within the NYFHT in January of 2019; there were 86 FPs at the time of the study. Two reminders containing a link to the research survey were sent to participants at three and six weeks after the initial invitation. An additional waiting period of three weeks was included after the last reminder was sent to allow for any delayed participation.

### Data analysis

Raw data were compiled using Qualtrics. Demographic and quantitative data were collated in tables with percentages. Qualitative responses were thematically analyzed using inductive codes to group similar responses together. These codes were developed independently by C.C. and M.A. and compared across these researchers. When differences were apparent, rationale was shared and discussed and one way forward was agreed upon. Respondents were excluded if they were not practicing within the NYFHT during the specified time period.

## Results

### Demographics

Forty-one physicians returned the survey. Two participants indicated that they were not a FP in North York and one did not specify. These three individuals were excluded, yielding 38 valid respondents (44% of FPs at NYFHT). Demographic data are presented in Table 1. Seventy-one percent of NYFHT FPs were females, while 76% of respondents were females. Ages ranged from younger than 30 years to older than 70 years, with the majority being between 30 and 49 years of age (*n*=24/38; 63.2%). Experience ranged from “one to five” years of practice to over 36 years of practice.

**Table 1.** Demographic Data of Survey Respondents

	Respondents (*n*=38)
Sex	Females: 29 (76.3%)
	Males: 8 (21.0%)
	Unspecified: 1 (2.6%)
Age in years	Under 30: 1 (2.6%)
	30–39: 11 (28.9%)
	40–49: 13 (34.2%)
	50–59: 5 (13.2%)
	60–69: 7 (18.4%)
	70+: 1 (2.6%)
Number of years in practice	1–5: 5 (13.2%)
	6–10: 7 (18.4%)
	11–15: 8 (21.0%)
	16–20: 4 (10.5%)
	21–25: 2 (5.3%)
	26–30: 3 (7.9%)
	31–35: 4 (10.5%)
	36+: 4 (10.5%)
	Unspecified: 1 (2.6%)

### Participation in the TPS program

Thirty-nine percent of NYFHT FPs participated in the TPS program. In our survey, half of the respondents (*n*=19/38; 50%) indicated that they enrolled in the TPS program. Reasons provided by respondents for not enrolling in the TPS Program (*n*=19/38 respondents; 50%) were varied. The most commonly provided reason was having either too many preexisting commitments or not enough time or energy (*n*=5/19; 26.3%).

The second most common reason was not being aware of or not recalling receiving the invitation (*n*=4/19; 21.0%). This was followed by being on leave from the practice (parental leave or sabbatical) (*n*=3/19; 15.8%); not receiving a notification or invitation about the program (*n*=3/19; 15.8%); and either forgetting or missing the deadline (*n*=2/19; 10.5%). One respondent (*n*=1/19; 5.3%) indicated “I perceived that I knew who in my practice was living in poverty. I use the tool when I think there is an issue. Most [of] my patients make more [money] than I do.”

### Self-identified screening behavior

#### Those who screened

Irrespective of enrollment in the TPS Program, the majority of respondents (*n*=31/38; 81.6%) reported screening their patients for poverty using the evidence-based question (detailed in Table 2). Among those not enrolled in the program, 84.2% (*n*=16/19) indicated that they screened their patients.

**Table 2.** Self-Identified Screening Behavior

	Respondents who answered “yes” as opposed to “no”
Enrolled in the TPS Program within the NYFHT	19/38 (50%)
Screened patients using the evidence-based question (regardless of enrollment in the TPS Program)	31/38 (81.6%)
Among those enrolled in the TPS Program, those who indicated screening patients using the evidence-based question	15/19 (78.9%)
Among those not enrolled in the TPS Program, those who indicated screening patients using the evidence-based question	16/19 (84.2%)
Referred patients to a case worker for issues related to income/poverty	25/38 (65.8%)
From those who indicated they referred patients to a case worker for issues related to income/poverty, if this was a result of using the evidence-based question	17/25 (68%)

NYFHT, North York Family Health Team; TPS, Targeted Poverty Screening.

Respondents who indicated they screened for poverty (*n*=31/38; 81.6%) provided a variety of reasons, including screening being part of an electronic medical record (EMR) template or part of the physician's routine (*n*=7/31; 22.6%); perceived importance of screening for poverty/perceived potential to help/poverty as a SDH/important for prevention and health promotion (*n*=6/31; 19.4%); being taught to screen during training/NYFHT meeting (*n*=2/31; 6.5%); and screening for poverty being evidence-based (*n*=1/31; 3.2%). However, a plurality of respondents who indicated that they screened for poverty did not provide a reason as to why (*n*=15/31; 48.4%).

A majority (*n*=25/38; 65.8%) indicated that they referred patients to a case worker for issues related to income/poverty (Table 2). Of those who indicated that they did refer to a case worker, the majority (*n*=17/25; 68%) indicated that this was a result of screening.

#### Those who did not screen

Regardless of enrollment in the TPS Program, 18.4% (*n*=7/38) of respondents indicated that they did not screen patients using the evidence-based question. Four of these respondents who indicated that they did not screen for poverty were enrolled in the TPS Program.

Among respondents who did not screen (*n*=7/38; 18.4%), the reasons for not screening patients were “the [screening question] is in [already] the periodic health review [template],” or “a nurse or locum did the screening as part of a periodic health review” (i.e., patient was screened, but not by physician completing the survey; *n*=3; 42.95%); forgot (*n*=2; 28.6%); time constraints/feel uncomfortable asking (*n*=1; 14.3%); and feeling as though they know their patients well (*n*=1; 14.3%).

#### Suggestions put forward to make poverty screening easier/acceptable/feasible

Respondents also provided suggestions for making poverty screening easier/acceptable/feasible for FPs, outlined in Table 3.

**Table 3.** Suggestions Provided by Respondents for Making Poverty Screening Easier/Acceptable/Feasible for Family Physicians

Suggestions for building screening into templates and questionnaires
Place a prompt for screening in the physician's PHR template. Place reminders in more than one place (e.g., under “Social History” section of CPP and in the PHR template). Include screening questions in pre-appointment questionnaires (on a tablet or paper, or via e-mail). NB: Physicians should be mindful that patients may not be comfortable answering these questions in the waiting room with others around.
Alternative phrasing and questions
Use softer question prefaces like “we are examining the link between finances and health. It's suggested that I ask you about…” Asking about patients' housing situations, work situation, or enrolment of children in extracurricular activities. This may help uncover some financial difficulties.
Utilization of allied health care team members^a^
Arrange for allied health care team members to perform the screening. Post signs in the waiting room that explain the role of case managers, then ask patients if they would be interested in learning more once in the privacy of the examination room.

^a^ Please note: most patients are not seen routinely by allied health professionals.

CPP, Cumulative Patient Profile; NB, nota bene; PHR, Periodic Health Review.

Some noteworthy comments from respondents were as follows: “I want more information on helpful resources that we can distribute to patients, aside from just referring them to a Social Worker,” and “if I thought it would fix the problem I am certain I would screen more aggressively.” While these comments are from two respondents, they do point to a wider issue around the next step(s) following poverty screening.

## Discussion

To better understand the screening practices of FPs in one area, this exploratory study targeted FPs in North York who were invited to the initial TPS Program. This study determined that irrespective of enrollment in the TPS Program, 81.6% of respondents elected to screen their patients for poverty using the evidence-based question. Among those not enrolled in the program, 84.2% of respondents indicated that they screened their patients for poverty and 15.8% indicated they did not. These finding contrast findings from other studies, which determined that rates of screening are low.^10–13^ However, caution should be used when interpreting this figure as the study sought to collate responses from FPs practicing within the NYFHT.

Overall, given the positive result of the majority of respondents indicating that poverty screening was in fact the reason for referring patients to a case worker, further establishing and promoting TPS Programs is suggested. We conclude that (1) barriers faced by FPs in poverty screening can be mitigated, (2) there is a need to integrate screening into routines and normalize the activity, and (3) there is a need for enhanced training to support patients of lower socioeconomic status.

### Barriers faced by FPs in poverty screening can be mitigated

With the benefits of poverty screening being clear, it is imperative to explore why some participants elected not to undertake poverty screening. Reasons cited in the literature for limited poverty screening include insufficient education (formal and informal); low self-efficacy; lack of specialized and well-equipped communities; time constraints; and disproportionate remuneration.^10,11^ In addition, negative attitudes toward low-income populations, or viewing their social problems as “outside the physician's scope” have been noted.^12^

Among respondents who indicated that they did not screen patients for poverty, the response with the highest frequency was that the prompt was already in the periodic health review (PHR) or that a nurse or locum did the screening as part of the PHR or “stamp” for check-up, that is, the patient ultimately was screened. Other responses included “forgot”; “time constraints/feel uncomfortable asking”; and “feel I know patients well.” It is interesting that Brcic's findings run contrary to this; patients understand the need to ask about income and physicians should feel comfortable doing so.^9^

We note that perceived barriers can be mitigated. FPs can place reminders and can delegate the majority of screening to other health care workers or schedule longer appointments. FPs can be coached and encouraged on how to become familiar with asking about a patient's finances.

Poverty screening consists of asking one evidence-based question (with the option of asking two additional questions about food and housing security). This should not be particularly time-consuming for FPs. Another respondent indicated, “I perceived that I knew who in my practice was living in poverty. I use the tool when I think there is an issue. Most [of] my patients make more than I do.” Given the fact that 20% of Ontario's population is below the poverty line,^14^ awareness of the prevalence of poverty should be raised among FPs. Some respondents were either unaware or unavailable at the time of training, this points to the need to raise awareness among FPs about what poverty screening is.

Furthermore, it would be reasonable for physicians, particularly those in an affluent area, to wish for a tool that was not just sensitive, but also specific for finding persons living in poverty. Brcic et al.^9^ developed a poverty screening tool. The single question “Do you have trouble making ends meet at the end of the month” was used here as well, and was found to have a sensitivity of 98% for being below the calculated “Low Income Cut-Off” (LICO) for respondents (Statistics Canada definition).

Combining this question with (1) “In the past year, was there any day when you or anyone in your family went hungry because you did not have enough money for food?” and (2) “In the last month, have you slept outside, in a shelter, or in a place not meant for sleeping?” resulted in a specificity of 94%. Of those who were below the LICO and gave feedback, 85% believed poverty case-finding was “somewhat or very” important, and 67% were “very or somewhat comfortable speaking to their family physician about poverty-related issues.”^9^ That is, acceptability was high.

Further, being “uncomfortable” does not translate to unwillingness to discuss. Other uncomfortable topics must be, and are, confronted in-office on a daily basis. There are two important conclusions here. First, screening does not have to be time-consuming. Second, there can be a high degree of acceptability to screening for sensitive issues if it is done in a sensitive manner, as patients themselves find it an important topic to discuss.

### There is a need to integrate screening into routines and normalize the activity

Irrespective of their enrollment in the TSP (i.e., enrolled or not), 81.6% of respondents indicated that they screened patients using the evidence-based question. When asked why they screened, 22.6% respondents indicated that it was due to the question being part of their periodic health examination or review template, part of a physician's routine, or EMR. The second most commonly cited reason was the perceived importance of screening for poverty/perceived potential to help/poverty as a SDH/important for prevention and health promotion.

This suggests that integrating screening into routine, and normalizing the activity, is an effective way to increase uptake of screening. It also suggests that using the importance of poverty as motivation for screening may be effective. Indeed, many of the suggestions for increasing screening rates focused on either normalizing the activity or implementing more reminders.

Three respondents alluded to the case-finding potential (i.e., prompts for poverty screening to identify those affected), which may indicate potential for a role for case-finding in a more affluent population.

The feasibility of screening for lifestyle risk factors via case-finding was evaluated in a Canadian survey study that used a convenience sample in a family practice. The authors used the Case-finding Health Assessment Tool (CHAT), which was developed in New Zealand and previously validated to identify “lifestyle and mental health risk factors” (e.g., tobacco, alcohol, and other drug use; problem gambling; abuse). Moreover, they assessed “Rates of completion; positive responses to and wanting help with identified lifestyle and mental health risk factors; rates of objections to any questions; and positive and negative comments about the CHAT by participating physicians and patients.”

Out of 107 participants who completed the feedback form, only two participants objected to a single question (no other questions received any objections; that is, acceptability of case-finding was high in this study), and 35% of respondents requested same-day help with modifying a risk factor. This particular screening test can be completed in under five minutes. (Screening for poverty can be even shorter).^15^

### There is a need for enhanced training to support patients of lower socioeconomic status

With respect to the two respondents who pointed to a wider issue around the next step following poverty screening, particular consideration should also be afforded for individuals' own capabilities to promote more equitable and just action.^16^ Perhaps a bigger push for continuing medical education (CME) is necessary. Coupled with the knowledge that 26.3% of respondents who did not participate in the TPS program cited “too many preexisting commitments/not enough time or energy” as being the reason why, having an established CME credit on this topic may work to reduce the burden FPs may feel to learn about this issue independently and the feeling that they are not well-equipped.

### Study limitations

First, there may be bias in considering which FPs elected to partake in the study. This is a potential limitation, as this may result in a more favorable picture for engaging in poverty screening among FPs who were not enrolled in the TPS and may not reflect the behavior of FPs who did not complete the survey.

Second, there may be social desirability bias at play, where FP respondents may feel inclined to answer in a more positive way. However, with the anonymity of responses in the survey, we do not believe that this would have a large role in skewing responses in one direction (e.g., respondents feeling they need to answer “yes”).

Third, because this was a survey conducted in Toronto, Canada, the findings may not be generalizable to other regions where the FP culture may be different.

Finally, while FP engagement in poverty screening was assessed, these findings could not be compared to actual poverty screening behaviors, as these data were not collected in this study. However, for further information on poverty screening behaviors following from the implementation of the TPS Program, please refer to the article by Wintemute et al.^13^

## Conclusion

There is increasing recognition that poverty is “the result of a toxic combination of poor social policies and programs, unfair economic arrangements, and bad politics.”^17^ It may be seen as legitimate to ask whether FP engagement distracts from the responsibility of governments and society, and further, from the duty of the FP to provide health care.^18^ We argue that providing health care is the central role of a FP; therefore considering health holistically is crucial to addressing ill health. FPs may also draw on gleanings from undertaking poverty screening, engage in conversations, and utilize their authoritative voice in health to advocate for structural changes and policies that improve health, such as the Health in All Policies approach.^18^

In addition, with respect to the criteria for screening programs discussed in the introduction, particularly, “There should be scientific evidence of screening program effectiveness”,^7^ we recommend further attention be paid to collating evidence on effectiveness. Because, at present, the effectiveness of physicians and hospitals addressing the SDH, including income and poverty (classified differently depending on the categorization of the SDH), is unknown.^18^ As such, potential next steps include studying the impact of screening (e.g., studying health outcomes and/or patient experiences of those referred to a case worker) to elucidate whether or not screening can indeed lead to amelioration of any of the myriad health effects of poverty.

If it is supported, TPS Programs can be scaled-up to promote poverty screening. In addition, poverty screening can be integrated into routines to normalize the activity. Similarly, enhanced training should be provided to FPs through CME. This could touch on concrete strategies to better support patients; or more widely around different conceptualizations of health equity and the implications for action (e.g., striving for a baseline level of health for all versus in addition to reducing inequality^19,20^) to aid in FP understanding and perhaps garner interest.

## Abbreviations Used

CHAT: Case-finding Health Assessment Tool
CME: continuing medical education
CPP: Cumulative Patient Profile
EMR: electronic medical record
FP: family physician
NB: nota bene (latin for “note well”)
NYFHT: North York Family Health Team
PHR: periodic health review
SDH: social determinant of health
TPS: Targeted Poverty Screening

## Appendix A1

(1) Age:
(2) Sex:
(3) Approximately how many years have you been in practice?:
(4) Are you a Family Physician within North York Family Health Team? Y/N
(5) Were you actively treating patients within NYFHT between June 01, 2017 and November 30, 2017? Y/N
(6) Did you enroll in (i.e., sign up, receive orientation or coaching around) the Targeted Poverty Screening program? Y/N
(7) If your answer to Question (3) was “No,” please provide any reasons for why you did not enroll?
(8) Regardless of whether or not you formally enrolled in the program, did you screen patients for poverty using the evidence-based question “Do you have difficulty making ends meet at the end of the month?” during the relevant time period (June 01, 2017 – November 30, 2017)? Y/N
(9) If your answer to Question (5) was “No,” could you please provide any/all reasons for why you did not screen?
(10) If you were not enrolled in the Targeted Poverty Screening program, but did in fact screen, why did you do this?
(11) Did you refer patients to a Case Worker for issues related to income/poverty during the above-specified time period? Y/N
(12) If yes to Question (8), was it as a result of screening for poverty using the evidence-based question, as above? Y/N
(13) Do you have any ideas for making poverty screening more easy/acceptable/feasible for Family Physicians?
(14) Are you aware of any screening tools for poverty/low income? If so, what are they?
(15) Please provide your estimate of the percentage of patients within your practice that would answer “yes” to the question “Do you have difficulty making ends meet at the end of the month?”
